# Thermodynamics and Cosmic Censorship Conjecture in Kerr–Newman–de Sitter Black Hole

**DOI:** 10.3390/e20110855

**Published:** 2018-11-07

**Authors:** Bogeun Gwak

**Affiliations:** Department of Physics and Astronomy, Sejong University, Seoul 05006, Korea; rasenis@sejong.ac.kr

**Keywords:** black hole, thermodynamics, cosmic censorship conjecture

## Abstract

We investigate the laws of thermodynamics and the validity of the cosmic censorship conjecture in the Kerr–Newman–de Sitter black hole under charged particle absorption. Here, the black hole undergoes infinitesimal changes because of the momenta carried by the particle entering it. The cosmic censorship conjecture is tested by whether the black hole can be overcharged beyond the extremal condition under absorption. The changes in the black hole violate the second law of thermodynamics. Furthermore, this is related to the cosmic censorship conjecture. To resolve this violation, we impose a reference energy of the particle at the asymptotic region based on the first law of thermodynamics. Under imposition of the reference energy, the absorption satisfies the laws of thermodynamics, and the extremal black hole cannot be overcharged. Thus, the cosmic censorship conjecture is valid under the absorption.

## 1. Introduction

The accelerated expansion of our universe as evidenced by the data obtained from cosmological evolution or supernovae [1,2,3] suggests the presence of a small positive cosmological constant in Einstein gravity. The cosmological constant provides a negative pressure in the universe, thereby affecting the configurations of the solutions of the Einstein field equations, such as black holes. In particular, the black hole solution with a positive cosmological constant is called the de Sitter (dS) black hole. The physics of black holes has become important for studying the observations of GW150914, GW151226, and GW170104 by using the Laser Interferometer Gravitational-Wave Observatory (LIGO) [4,5,6]. The dS black hole is also considered to be important for the Higgs potential in a high-energy regime after the discovery of the Higgs particle [7,8]. Studies concerning the Higgs potential suggest that the present universe may be metastable, and could thus decay into true vacua in finite time. The lifetime of the metastable vacuum is large enough to cover the age of the present universe because of a large barrier [9,10,11]. However, inhomogeneities are generated from gravitational impurities, for example, the dS black holes can reduce the barrier and thus the lifetime up to millions of Planck times [12,13,14].

Black holes have a singularity within their horizon. At the singularity, causality may break down, making physics unpredictive. To avoid this situation, the cosmic censorship conjecture suggests that the singularity should always be covered by an event horizon [15]. Hence, the cosmic censorship conjecture should be valid for dS black holes. The validity of this conjecture has been mainly investigated in terms of the possibility of overspinning or overcharging a black hole beyond its extremal limits by means of an external particle or field. No horizon exists in an overspun or overcharged black hole. Hence, the cosmic censorship conjecture is not true in these cases. In asymptotically flat spacetime, the conjecture is valid for the Kerr black hole under particle absorption [16]. A particle could cause a near-extremal Kerr black hole to overspin [17,18,19]. However, if the self-force is considered, the conjecture is still valid [20,21,22,23]. Similarly, the cosmic censorship conjecture has also been investigated in charged black holes [24,25,26,27,28,29], black holes with a negative cosmological constant [30,31,32,33,34,35], and in lower [36,37,38] and higher dimensions [39,40,41,42,43,44].

The thermodynamics of a black hole is an important aspect of the physics of such a gravitational system. The mass of the black hole consists of reducible and irreducible masses. The reducible mass (e.g., the rotational and electric energies) can be decreased by adding a particle. For instance, rotational energy can be extracted from a black hole through the Penrose process [45,46]. However, the irreducible mass, which is distributed on the horizon of the black hole [47], always increases upon adding a particle [48,49]. As a result, the area of the black hole always increases. This irreversible increase is similar to that of entropy in thermodynamics. From this similarity, the entropy of a black hole has been shown to be proportional to its area—a relation known as the Bekenstein–Hawking entropy [50,51]. In addition, the black hole can release energy through quantum effects. From this radiation, the Hawking temperature of the black hole can be defined in terms of the surface gravity [52,53]. By using these thermodynamic variables, the black hole can be treated as a thermal system, and the laws of thermodynamics for the black hole can be defined. Various aspects of its thermodynamics are now studied [54,55,56].

The distinct property of the dS black hole is the location of its inner and outer horizons. However, outside the outer horizon, the spacetime also has a cosmological horizon. As a result, the observable range is confined between the outer and cosmological horizons. The cosmological horizon has similar properties to the black hole horizons, and the laws of thermodynamics can be defined for it as well. Hence, two temperatures exist in the spacetime, that is, those defined on the outer and cosmological horizons, and the system is not in thermal equilibrium. To describe the system, an effective temperature was introduced [57,58,59], and the gravitational entropy is considered as the sum of the areas of the outer and cosmological horizons [60,61]. In addition, in the dS spacetime, the cosmological constant can be considered as a pressure term in the effective first law of thermodynamics [62,63,64]. Although the thermodynamics of the dS spacetime has been investigated in various studies [65,66,67,68,69,70,71], it is still an interesting topic of study.

Herein, we investigate the relation between the laws of thermodynamics and the cosmic censorship conjecture in the Kerr–Newman–de Sitter (KNdS) black hole under the condition of charged particle absorption. In addition, the equations of motion of the charged particle should ensure that the laws of thermodynamics are satisfied on the outer horizon because the black hole changes infinitesimally owing to the particle. Next, we show that the outer horizon still exists in the extremal black hole under charged particle absorption. Therefore, the cosmic censorship conjecture is valid for a four-dimensional black hole with a positive cosmological constant, provided that the laws of thermodynamics are satisfied.

This paper is organized as follows: Section 2 introduces the KNdS black hole and its thermodynamic properties; Section 3 shows that under charged particle absorption, the equations of particle motion should be modified to satisfy the laws of thermodynamics, and we obtain the changes in the black hole in terms of the conserved quantities of the particle; Section 4 proves that the cosmic censorship conjecture is valid for an extremal KNdS black hole; and finally, Section 5 briefly summarizes our results.

## 2. Kerr–Newman–de Sitter Black Holes

The KNdS black hole is a solution to Einstein gravity coupled with a gauge field and with a positive cosmological constant Λ [72]. The KNdS black hole is described as follows:

(1) $$\begin{array}{cc} ds^{2} & =-\frac{\Delta_{r}}{\rho^{2}}{\left(dt-\frac{a\sin^{2}\theta }{\Xi }d\phi \right)}^{2}+\frac{\rho^{2}}{\Delta_{r}}dr^{2}+\frac{\rho^{2}}{\Delta_{\theta }}d\theta^{2}+\frac{\Delta_{\theta }\sin^{2}\theta }{\rho^{2}}{\left(a\;dt-\frac{r^{2}+a^{2}}{\Xi }d\phi \right)}^{2}\;, \end{array}$$

$$\begin{array}{cc} \Delta_{r} & =\left(r^{2}+a^{2}\right)\left(1-\frac{1}{3}\Lambda r^{2}\right)-2Mr+Q^{2}\;,\;\Delta_{\theta }=1+\frac{1}{3}\Lambda a^{2}\cos^{2}\theta \;, \end{array}$$

$$\begin{array}{cc} \rho^{2} & =r^{2}+a^{2}\cos^{2}\theta \;,\;\Xi =1+\frac{1}{3}\Lambda a^{2}\;, \end{array}$$

where *M*, *a*, and *Q* are the mass, spin, and electric charge parameters, respectively. The gauge potential *A* is defined as [73,74]

(2) $$\begin{array}{c} A=-\frac{Qr}{\rho^{2}}\left(dt-\frac{a\sin^{2}\theta }{\Xi }d\phi \right)\;. \end{array}$$

Note that the Kerr black hole with Λ=0 and Q=0 is included as a specific case of the Quevedo–Mashhoon solution [75,76,77]. The mass, angular momentum, and electric charge of the KNdS black hole are $M_{B}$, $J_{B}$, and $Q_{B}$, respectively, and are defined as [63,73]:

(3) $$\begin{array}{c} M_{B}=\frac{M}{\Xi^{2}}\;,\;J_{B}=\frac{Ma}{\Xi^{2}}\;,\;Q_{B}=\frac{Q}{\Xi }\;. \end{array}$$

For a positive cosmological constant, an extra allowed solution of $\Delta_{r}=0$ exists, which is the cosmological horizon $r_{c}$ located outside of the outer horizon $r_{h}$. Thus, the observable region is limited to the space between the two surfaces, $r_{h}<r<r_{c}$. On these surfaces, the properties of the black hole can be defined in a similar way. The angular velocity on the outer horizon is [73]:

(4) $$\begin{array}{c} \Omega_{h}=\frac{a\Xi }{r_{h}^{2}+a^{2}}\;. \end{array}$$

Moreover, the Hawking temperature and electric potential on the outer horizon are respectively defined as

(5) $$\begin{array}{c} T_{H}=\frac{r_{h}\left(1-\frac{\Lambda a^{2}}{3}-\frac{a^{2}+Q^{2}}{r_{h}^{2}}-\Lambda r_{h}^{2}\right)}{4\pi \left(r_{h}^{2}+a^{2}\right)}\;,\;\Phi_{H}=\frac{r_{h}Q}{r_{h}^{2}+a^{2}}\;. \end{array}$$

The area of the outer horizon $A_{H}$ is related to the Bekenstein–Hawking entropy $S_{BH}$,

(6) $$\begin{array}{c} S_{BH}=\frac{1}{4}A_{H}=\frac{\pi (r_{h}^{2}+a^{2})}{\Xi }\;. \end{array}$$

The first law of thermodynamics is given as [63,64,65,66,67]:

(7) $$\begin{array}{c} \delta M_{B}=T_{H}\delta S_{BH}+\left(\Omega_{h}-\Omega_{0}\right)\delta J_{B}+\Phi_{H}\delta Q_{B}\;, \end{array}$$

where the reference angular velocity is denoted as $\Omega_{0}$. Two main possible choices can be considered for the reference angular velocity. The first is $\frac{a\Lambda }{3}$ based on the rotation on the spacetime boundary r→∞ [78]. This choice is consistent with the anti-de Sitter (AdS) case [66,67], even though the boundary is not at an observable region. The other possibility, $\frac{a\Xi }{r_{c}^{2}+a^{2}}$, is based on the cosmological horizon [71] and hence removes its rotation. For consistency, the first law of thermodynamics on the cosmological horizon should be written in terms of the same reference angular velocity:

(8) $$\begin{array}{c} \delta M_{B}=-T_{c}\delta S_{c}+\left(\Omega_{c}-\Omega_{0}\right)\delta J_{B}+\Phi_{c}\delta Q_{B}\;, \end{array}$$

where the entropy $S_{c}$, angular velocity $\Omega_{c}$, electric potential $\Phi_{c}$, and temperature $T_{c}$ defined on the cosmological horizon are

(9) $$\begin{array}{c} S_{c}=\frac{\pi (r_{c}^{2}+a^{2})}{\Xi }\;,\;\Omega_{c}=\frac{a\Xi }{r_{c}^{2}+a^{2}}\;,\;\Phi_{c}=\frac{r_{c}Q}{r_{c}^{2}+a^{2}}\;,\;T_{c}=\frac{r_{c}\left(1-\frac{\Lambda a^{2}}{3}-\frac{a^{2}+Q^{2}}{r_{c}^{2}}-\Lambda r_{c}^{2}\right)}{4\pi \left(r_{c}^{2}+a^{2}\right)}\;. \end{array}$$

The minus sign of the temperature $T_{c}$ in Equation (8) is necessary for the first term to be positive because the sign of the surface gravity is negative on the cosmological horizon [63,64].

The metric in Equation (1) becomes a well-known Kerr–Newman (KN) black hole at Λ=0, which is asymptotically flat. Here, there are only two solutions to $g^{rr}=0$ corresponding to the inner and outer horizons, $r_{i}$ and $r_{h}$. They are located at

(10) $$\begin{array}{c} r_{i}=M-\sqrt{M^{2}-a^{2}-Q^{2}},\;r_{h}=M+\sqrt{M^{2}-a^{2}-Q^{2}}, \end{array}$$

where the horizons exist in $M^{2}>a^{2}+Q^{2}$. The extremal condition is $M^{2}=a^{2}+Q^{2}$, where the inner and outer horizons are coincident. Overcharged to $M^{2}<a^{2}+Q^{2}$, the solution does not have a horizon. Hence, the central region of the spacetime is no longer covered by the horizon. Outside of the outer horizon, the surface of the infinite red shift is located in the KN black hole. Given by $g_{tt}=0$ with a positive sign solution, the surface has a radius of

(11) $$\begin{array}{c} r_{s}=M+\sqrt{M^{2}-a^{2}\cos^{2}\theta -Q^{2}}. \end{array}$$

The region between the two surfaces of $r_{h}$ and $r_{s}$ is called the ergosphere. The location of the singularity is indicated by the Kretschmann scalar $R^{\mu \nu \rho \sigma }R_{\mu \nu \rho \sigma }$. The singularity is then at $\rho^{2}=0$ satisfied by r=0 and θ=π/2. In Cartesian coordinates, r=0 and θ=π/2 become

(12) $$\begin{array}{c} x^{2}+y^{2}=a^{2},\;z=0, \end{array}$$

which is a ring with radius *a*. Thus, the ring singularity exists in the KN black hole. For the asymptotic observer, the singularity is covered by the outer horizon. However, if the black hole is overcharged beyond the extremal condition, the horizons will disappear, and the singularity will be exposed to the asymptotic observer. We will investigate whether the black hole can be the naked singularity by charged particle absorption.

## 3. Laws of Thermodynamics under Charged Particle Absorption

When a particle is absorbed by a KNdS black hole, the black hole experiences infinitesimal changes on its properties because of the conserved quantities of the particle. Based on these changes, we will prove the validity of the cosmic censorship conjecture for the KNdS black hole. In the proof, the choice of the reference angular velocity $\Omega_{0}$ plays an important role in satisfying the laws of thermodynamics. To find the relation between the conserved quantities of the particle and the black hole properties, the first-order equations of motion of the particle should be determined using the Hamilton–Jacobi method [72,79,80,81] based on the separation of variables. We can then define the particle energy in terms of its charges and introduce an exact relation that determines how much energy and particle charges are absorbed by the black hole. The Hamiltonian and Hamilton–Jacobi actions for a particle with mass *m*, momentum $p_{\mu }$, and electric charge *e* are written as

(13) $$\begin{array}{c} H=\frac{1}{2}g^{\mu \nu }\left(p_{\mu }-eA_{\mu }\right)\left(p_{\nu }-eA_{\nu }\right)\;,\;S=\frac{1}{2}m^{2}\lambda -Et+L\phi +S_{r}\left(r\right)+S_{\theta }\left(\theta \right)\;, \end{array}$$

where the conserved quantities *E* and *L* correspond to the energy and angular momentum of the particle, respectively, and the electric charge *e* of the particle is coupled with the gauge potential. From Equation (13), the Hamiltonian equation can be written as

(14) $$\begin{array}{cc} -m^{2}= & -\frac{1}{\Delta_{r}\rho^{2}}{\left(\left(r^{2}+a^{2}\right)\left(E-\frac{eQr}{\rho^{2}}\right)-a\Xi \left(L-\frac{aeQr\sin^{2}\theta }{\rho^{2}\Xi }\right)\right)}^{2}+\frac{\Delta_{r}}{\rho^{2}}{\left(\partial_{r}S_{r}\right)}^{2}+\frac{\Delta_{\theta }}{\rho^{2}}{\left(\partial_{\theta }S_{\theta }\right)}^{2}\\ & +\frac{1}{\Delta_{\theta }\rho^{2}}{\left(a\sin\theta \left(E-\frac{eQr}{\rho^{2}}\right)-\Xi \csc\theta \left(L-\frac{aeQr\sin^{2}\theta }{\rho^{2}\Xi }\right)\right)}^{2}\;, \end{array}$$

which is a separable equation. Thus, we can find two separate equations depending only on *r* and ϕ by using a constant K. All the geodesic equations of the particle can be obtained from Equation (14). However, we only need the geodesics in the radial and θ directions to determine the energy of the particle:

(15) $$\begin{array}{cc} p^{r}\equiv \frac{dr}{d\lambda } & =\frac{\Delta_{r}}{\rho^{2}}\sqrt{R(r)}\;,\;R\left(r\right)=\frac{K}{\Delta_{r}}+\frac{1}{\Delta_{r}^{2}}{\left(\left(r^{2}+a^{2}\right)E-a\Xi L-eQr\right)}^{2}-\frac{m^{2}r^{2}}{\Delta_{r}}\;, \end{array}$$

$$\begin{array}{cc} p^{\theta }\equiv \frac{dr}{d\lambda } & =\frac{\Delta_{\theta }}{\rho^{2}}\sqrt{\Theta (\theta )}\;,\;\Theta \left(\theta \right)=-\frac{K}{\Delta_{\theta }}-\frac{\sin^{2}\theta }{\Delta_{\theta }^{2}}{\left(aE-\frac{\Xi L}{\sin^{2}\theta }\right)}^{2}-\frac{m^{2}a^{2}\cos^{2}\theta }{\Delta_{\theta }}\;, \end{array}$$

where $\frac{\partial r}{\partial \lambda }=\dot{r}\equiv p^{r}$ and $\frac{\partial \theta }{\partial \lambda }=\dot{\theta }\equiv p^{\theta }$ are the components of the momentum in the radial and θ directions, respectively. We removed the separation constant K in Equation (15) to obtain the energy for the given $p^{r}$ and $p^{\theta }$. We then determined the following equation for the energy in terms of the position and given charges:

(16) $$\begin{array}{c} \alpha E^{2}+2\beta E+\gamma =0\;, \end{array}$$

$$\begin{array}{c} \alpha =\frac{-{\left(r^{2}+a^{2}\right)}^{2}\Delta_{\theta }+a^{2}\Delta_{r}\sin^{2}\theta }{\Delta_{r}\Delta_{\theta }\rho^{4}}\;,\;\beta =\frac{eQr\left(r^{2}+a^{2}\right)\Delta_{\theta }+aL\left(-\Delta_{r}+\left(r^{2}+a^{2}\right)\Delta_{\theta }\right)\Xi }{\Delta_{r}\Delta_{\theta }\rho^{4}}\;,\\ \gamma =\frac{{\left(p^{r}\right)}^{2}\rho^{4}-{\left(eQr+aL\Xi \right)}^{2}}{\Delta_{r}\rho^{4}}+\frac{{\left(p^{\theta }\right)}^{2}+\frac{L^{2}\Xi^{2}\csc^{2}\theta }{\rho^{4}}}{\Delta_{\theta }}+\frac{m^{2}}{\rho^{2}}\;. \end{array}$$

The particle is future-forwarding. Hence, the positive sign should be chosen for the solution of Equation (16) [48,49]. We suppose that the particle is absorbed by the black hole when it crosses the outer horizon. Thus, Equation (16) for the energy should be solved at the position of the outer horizon (i.e., $r=r_{h}$). Therefore, we obtain the following expression:

(17) $$\begin{array}{c} E_{h}=\frac{a\Xi }{r_{h}^{2}+a^{2}}L+\frac{r_{h}Q}{r_{h}^{2}+a^{2}}e+\frac{\rho_{h}^{2}}{r_{h}^{2}+a^{2}}\left|p^{r}\right|\;,\;\rho_{h}^{2}=r_{h}^{2}+a^{2}\cos^{2}\theta \;, \end{array}$$

where $\rho^{2}{|}_{r=r_{h}}=\rho_{h}^{2}$. In the asymptotically flat limit Λ→0, the energy in Equation (17) is equal to that of the Kerr–Newman black hole. In addition, if e→0, we obtain the energy of the Kerr black hole [41,48,49]. Note that the KN black hole has repulsive regions located inside or outside the outer horizon [82]. When a charged particle is absorbed by the KNdS black hole, the mass, angular momentum, and electric charge of the particle will be conserved in the spacetime because of the respective conservation laws. Thus, the conserved charges of the particle can be transferred to the black hole without loss. Consequently, we assume that the energy, angular momentum, and electric charge of the particle infinitesimally change to those of the black hole when the particle passes through the outer horizon. Thus,

(18) $$\begin{array}{c} E_{h}=\delta M_{B}\;,\;L=\delta J_{B}\;,\;e=\delta Q_{B}\;. \end{array}$$

Hence, by Equations (17) and (18), the change in the mass of the black hole can be written as

(19) $$\begin{array}{c} \delta M_{B}=\frac{a\Xi }{r_{h}^{2}+a^{2}}\delta J_{B}+\frac{r_{h}Q}{r_{h}^{2}+a^{2}}\delta Q_{B}+\frac{\rho_{h}^{2}}{r_{h}^{2}+a^{2}}\left|p^{r}\right|\;,\;\rho_{h}^{2}=r_{h}^{2}+a^{2}\cos^{2}\theta \;, \end{array}$$

which is a constraint between the variations in the mass, angular momentum, and electric charge of the black hole arising from the charges of the absorbed particle. Before testing the cosmic censorship conjecture, we should check whether Equation (19) satisfies the second law of thermodynamics because particle absorption is an irreversible process, and thus the entropy of the black hole should increase. The change in the Bekenstein–Hawking entropy is obtained from varying Equation (6) with respect to parameters *M*, *a*, *Q*, and $r_{h}$:

(20) $$\begin{array}{c} \delta S_{BH}=\delta \left(\frac{\pi (r_{h}^{2}+a^{2})}{\Xi }\right)=\frac{2\pi r_{h}}{1+\frac{a^{2}\Lambda }{3}}\delta r_{h}+\left(\frac{2a\pi }{1+\frac{a^{2}\Lambda }{3}}-\frac{2a\pi (r_{h}^{2}+a^{2})\Lambda }{3{\left(1+\frac{a^{2}\Lambda }{3}\right)}^{2}}\right)\delta a\;. \end{array}$$

Here, $\delta r_{h}$ is not an independent parameter, and can be removed based on the fact that the variation of $\Delta_{r}{|}_{r=r_{h}}=\Delta_{h}$ should vanish, because the position of the horizon is also infinitesimally shifted to $r_{h}+\delta r_{h}$:

(21) $$\begin{array}{cc} \delta \Delta_{h} & =-2r_{h}\delta M+2Q\delta Q+2a\left(1-\frac{r_{h}^{2}\Lambda }{3}\right)\delta a+\left(-2M-\frac{2}{3}r_{h}\left(r_{h}^{2}+a^{2}\right)\Lambda +2r_{h}\left(1-\frac{r_{h}^{2}\Lambda }{3}\right)\right)\\ & =0\;, \end{array}$$

from which the change in the position of the horizon $r_{h}$ is obtained as

(22) $$\begin{array}{c} \delta r_{h}=\frac{-3r_{h}\delta M+3Q\delta Q+a\left(3-r_{h}^{2}\Lambda \right)\delta a}{3M+r_{h}\left(-3+a^{2}\Lambda +2r_{h}^{2}\Lambda \right)}\;. \end{array}$$

After inserting Equation (22) into Equation (20), we remove δM by expressing it in terms of the variations of the particle charges. The value of δM is obtained through Equation (19) by using the relations given in Equation (3) as

(23) $$\begin{array}{c} \delta M=\frac{\frac{4aM\Lambda \delta a}{3{\left(1+\frac{a^{2}\Lambda }{3}\right)}^{3}}+\frac{a\left(1+\frac{a^{2}\Lambda }{3}\right)\left(\frac{M}{{\left(1+\frac{a^{2}\Lambda }{3}\right)}^{2}}-\frac{4a^{2}M\Lambda }{3{\left(1+\frac{a^{2}\Lambda }{3}\right)}^{3}}\right)\delta a}{r_{h}^{2}+a^{2}}+\frac{Qr_{h}\left(\frac{\delta Q}{\left(1+\frac{a^{2}\Lambda }{3}\right)}-\frac{2a^{2}Q\Lambda \delta a}{3{\left(1+\frac{a^{2}\Lambda }{3}\right)}^{2}}\right)\delta a}{r_{h}^{2}+a^{2}}+\frac{\rho_{h}^{2}\left|p^{r}\right|}{r_{h}^{2}+a^{2}}}{\frac{1}{{\left(1+\frac{a^{2}\Lambda }{3}\right)}^{2}}-\frac{a^{2}}{\left(r_{h}^{2}+a^{2}\right)\left(1+\frac{a^{2}\Lambda }{3}\right)}}\;. \end{array}$$

Finally, we obtain

(24) $$\begin{array}{cc} \delta S_{BH}= & f_{1}\delta a+f_{2}\delta Q+f_{3}\left|p^{r}\right|\;, \end{array}$$

$$\begin{array}{cc} f_{1}= & \frac{54a\pi \Lambda (a^{4}M+2a^{2}r_{h}^{2}-2\left(a^{2}+Q^{2}\right)r_{h}^{3}+5Mr_{h}^{4}-2r_{h}^{5})}{{\left(3+a^{2}\Lambda \right)}^{2}\left(-3r_{h}^{2}+a^{4}\Lambda \right)\left(3M+r_{h}\left(-3+a^{2}\Lambda +2r_{h}^{2}\Lambda \right)\right)}\\ & +\frac{36a\pi \Lambda r_{h}\left(a^{6}+r_{h}^{6}+a^{4}r_{h}\left(-2M+r_{h}\right)+a^{2}\left(-Q^{2}r_{h}^{2}+r_{h}^{4}\right)\right)\Lambda -12\pi a^{5}r_{h}^{3}\left(a^{2}+r_{h}^{2}\right)\Lambda^{3}}{{\left(3+a^{2}\Lambda \right)}^{2}\left(-3r_{h}^{2}+a^{4}\Lambda \right)\left(3M+r_{h}\left(-3+a^{2}\Lambda +2r_{h}^{2}\Lambda \right)\right)}\;, \end{array}$$

$$\begin{array}{cc} f_{2}= & \frac{18a^{2}\pi Qr_{h}\left(a^{2}+r_{h}^{2}\right)\Lambda }{{\left(3+a^{2}\Lambda \right)}^{2}\left(-3r_{h}^{2}+a^{4}\Lambda \right)\left(3M+r_{h}\left(-3+a^{2}\Lambda +2r_{h}^{2}\Lambda \right)\right)}\;, \end{array}$$

$$\begin{array}{cc} f_{3}= & \frac{6\pi r_{h}^{2}\left(3+a^{2}\Lambda \right)\rho_{h}^{2}}{\left(-3r_{h}^{2}+a^{4}\Lambda \right)\left(3M+r_{h}\left(-3+a^{2}\Lambda +2r_{h}^{2}\Lambda \right)\right)}\;. \end{array}$$

This shows that the second law of thermodynamics may be violated because we can freely choose the sign of δQ and δa by properly setting the electric charge and the angular momentum of the particle. For instance, if we set $p^{r}=0$ and the signs of δQ and δa opposite to those of $f_{1}$ and $f_{2}$, respectively, the change in the entropy $\delta S_{BH}$ would be negative. Thus, the area of the black hole would decrease, violating the second law of thermodynamics. Furthermore, such a black hole would violate the cosmic censorship conjecture. This is demonstrated in the Appendix A.

However, such a violation of the second law of thermodynamics can be prevented by properly choosing the reference angular velocity $\Omega_{0}$. This problem occurs because the metric of the KNdS black hole is still rotating in the asymptotic region, even if this region is not observable. Thus, when we measure the particle energy, we do not obtain the real value, but a value positively or negatively boosted by the coordinate rotation. Note that this coordinate effect is different from the dragging effect in the Kerr black hole (for which the rotating velocity tends to zero in the asymptotic region), and is only seen in rotating (A)dS black holes. The value of the reference energy should satisfy two conditions to remove the effect. The first is that it should not depend on any particular value of coordinate *r*, such as $r_{h}$ or $r_{c}$, because we are describing a black hole system that changes upon particle absorption. Hence, if the reference energy depended on $r_{h}$ or $r_{c}$, its value would correspond to a different location after the absorption. The other condition is consistency with the AdS case: the description for the KNdS black hole should also include the AdS black hole. Considering these points, there remains a mathematical choice. In the limit of r≫1, the solution for the energy of a massless particle obtained from Equation (16) reads

(25) $$\begin{array}{c} E_{\infty }=\frac{a\Lambda }{3}L+\sqrt{\frac{\Delta_{\theta }{\left(p^{r}\right)}^{2}}{\Xi }-\frac{\Lambda {\left(r^{2}p^{\theta }\right)}^{2}}{3\Xi }-\frac{\Lambda \Delta_{\theta }L^{2}}{3\sin^{2}\theta }}\;. \end{array}$$

The expression inside the square root in Equation (25) should be positive for the energy to be real and independent of the relative direction of rotation between the black hole and the particle (note that the term $r^{2}p^{\theta }$ has the same dimension as $p^{r}$ or *L*). However, the first term of Equation (25) depends on this relative direction of rotation. This term is produced by the rotation in the asymptotic region, and can be removed by assuming that the reference energy of the particle $E_{0}$ is precise.

(26) $$\begin{array}{c} E_{0}=\frac{a\Lambda }{3}L\;. \end{array}$$

This choice of $E_{0}$ implies that the reference angular velocity $\Omega_{0}$ equals $\frac{a\Lambda }{3}$ [83], which is consistent with the AdS case [44,66,67,73,84]. Therefore, the normalized energy of the particle *E* is obtained from Equations (17) and (26) as

(27) $$\begin{array}{c} E=E_{h}-E_{0}=\frac{a\left(1-\frac{\Lambda }{3}r_{h}^{2}\right)}{r_{h}^{2}+a^{2}}L+\frac{r_{h}Q}{r_{h}^{2}+a^{2}}e+\frac{\rho_{h}^{2}}{r_{h}^{2}+a^{2}}\left|p^{r}\right|\;. \end{array}$$

We calculated the energy in the asymptotic region to obtain the reference value. However, the redefined energy *E* can be measured by an observer inside the cosmological horizon. In addition, in the metric and Hamilton–Jacobi actions given by Equations (1) and (13), energy *E* can be measured by an observer with an angular velocity of $\frac{a\Lambda }{3}$ (where the values of *a* and Λ can be determined from the properties of the black hole and the angular momentum of the particle), such that we do not need information about the geometry outside of the cosmological horizon. Note that the redefined energy *E* corresponds to an energy obtained in the metric of Equation (1) transformed by [85]:

(28) $$\begin{array}{c} t\to T,\;\phi \to \Phi +\frac{1}{3}a\Lambda T. \end{array}$$

We now perform the exact same operation as in Equations (17)–(24), but replacing $E_{h}$ with *E*. We start by assuming that the conserved quantities of the particle in Equation (27) infinitesimally change the corresponding mass, angular momentum, and electric charge of the black hole:

(29) $$\begin{array}{c} E=\delta M_{B}\;,\;L=\delta J_{B}\;,\;e=\delta Q_{B}\;. \end{array}$$

The change in the mass of the black hole is then explicitly obtained from Equation (27) as:

(30) $$\begin{array}{c} \delta M_{B}=\frac{a\left(1-\frac{\Lambda }{3}r_{h}^{2}\right)}{r_{h}^{2}+a^{2}}\delta J_{B}+\frac{r_{h}Q}{r_{h}^{2}+a^{2}}\delta Q_{B}+\frac{\rho_{h}^{2}}{r_{h}^{2}+a^{2}}\left|p^{r}\right|\;. \end{array}$$

This is an expression that satisfies the second law of thermodynamics. The location of the outer horizon is also infinitesimally changed by $\delta r_{h}$. We determine the form of $\delta r_{h}$ by inserting Equation (30) into Equation (22) and obtaining

(31) $$\begin{array}{c} \delta r_{h}=-\frac{\Delta_{Mh}P_{Lh}+\Delta_{Jh}}{\Delta_{Dh}}L-\frac{\Delta_{Mh}P_{Qh}+\Delta_{Qh}}{\Delta_{Dh}}e-\frac{\Delta_{Mh}P_{Rh}}{\Delta_{Dh}}\left|p^{r}\right|\;, \end{array}$$

where

(32) $$\begin{array}{c} P_{Lh}=\frac{a\left(1-\frac{\Lambda }{3}r_{h}^{2}\right)}{r_{h}^{2}+a^{2}}\;,\;P_{Qh}=\frac{r_{h}Q}{r_{h}^{2}+a^{2}}\;,\;P_{Rh}=\frac{\rho_{h}^{2}}{r_{h}^{2}+a^{2}}\;, \end{array}$$

$$\begin{array}{c} \Delta_{Dh}=\frac{\partial \Delta_{h}}{\partial r_{h}}=-\frac{2}{3}\left(3M+r_{h}\left(-3+a^{2}\Lambda +2r_{h}^{2}\Lambda \right)\right)\;,\;\Delta_{Qh}=\frac{\partial \Delta_{h}}{\partial Q_{B}}=2Q\Xi \;, \end{array}$$

$$\begin{array}{c} \Delta_{Jh}=\frac{\partial \Delta_{h}}{\partial J_{B}}=-\frac{2a\Xi \left(-2Q^{2}\Lambda -3\Xi +r_{h}\Lambda \left(4M+r_{h}\Xi \right)\right)}{3M}\;, \end{array}$$

$$\begin{array}{c} \Delta_{Mh}=\frac{\partial \Delta_{h}}{\partial M_{B}}=\frac{2\Xi \left(-3Mr_{h}\Xi +a^{2}(-2Q^{2}\Lambda -3\Xi +r_{h}\Lambda \left(4M+r_{h}\Xi \right)\right)}{3M}\;. \end{array}$$

The contribution of the radial momentum to the variation of the position of the horizon is positive for a future-forwarding particle, whereas the contributions of the angular momentum and electric charge may be positive or negative. Hence, the change in the position of the horizon under particle absorption depends on the sign of these two parameters. However, the situation is quite different for the change in the entropy, and the area of the horizon can be shown to increase irrespective of the parameter values of the particle. The variation in the entropy is given as

(33) $$\begin{array}{cc} \delta S_{BH} & =\frac{\partial S_{BH}}{\partial M_{B}}\delta M_{B}+\frac{\partial S_{BH}}{\partial J_{B}}\delta J_{B}+\frac{\partial S_{BH}}{\partial r_{h}}\delta r_{h}\;, \end{array}$$

$$\begin{array}{cc} \frac{\partial S_{BH}}{\partial M_{B}} & =\frac{2a^{2}\pi \left(\left(r_{h}^{2}+a^{2}\right)\Lambda -3\Xi \right)}{3M}\;,\;\frac{\partial S_{BH}}{\partial J_{B}}=-\frac{2a\pi (\left(r_{h}^{2}+a^{2}\right)\Lambda -3\Xi )}{3M}\;,\;\frac{\partial S_{BH}}{\partial r_{h}}=\frac{2\pi r_{h}}{\Xi }\;, \end{array}$$

where there is no $\delta Q_{B}$ term because the entropy does not depend on $Q_{B}$. We then insert Equations (30) and (31) into Equation (33) and obtain the change in the entropy in terms of the radial momentum of the particle:

(34) $$\begin{array}{c} \delta S_{BH}=\frac{4\pi \rho_{h}^{2}}{\Delta_{Dh}}\left|p^{r}\right|\geq 0\;. \end{array}$$

Function $\Delta_{Dh}$ is positive for the KNdS black hole (except for an extremal black hole, for which it equals zero). Therefore, $\delta S_{BH}$ is always positive in the nonextremal case. Thus, for a nonextremal black hole, we can expect that the entropy always increases. Although we did not impose any information about the thermodynamics, the particle absorption process always increases the entropy of the black hole: a relation that is equivalent to the second law of thermodynamics. Hence, the outer horizon of a nonextremal black hole does not disappear because of the increase of the entropy under the particle absorption. Therefore, the cosmic censorship conjecture is still valid in the nonextremal case.

In Equation (34), the change in the entropy is proportional to the radial momentum of the particle. Thus, the radial momentum plays an important role in the change of the black hole. This is related to two types of energies in the black hole: irreducible and reducible masses. Irreducible mass $M_{ir}$ can be integrated out by removing the rotational and electric energies in Equation (30). Thus, we obtain

(35) $$\begin{array}{c} M_{ir}=\sqrt{\frac{r_{h}^{2}+a^{2}}{\Xi }}\;,\;\delta M_{ir}=\frac{4\rho_{h}^{2}}{M_{ir}\Delta_{Dh}}\left|p^{r}\right|\geq 0\;, \end{array}$$

which shows that $M_{ir}$ always increases upon particle absorption. Thus, the radial momentum of the particle adds to the irreducible mass of the KNdS black hole.

By inserting Equation (34) into Equation (30) to replace variable $p^{r}$ by $\delta S_{BH}$, we can derive the first law of thermodynamics. By identifying the coefficients of the variations with the temperature, angular velocity, and electric potential, we obtain

(36) $$\begin{array}{c} \delta M_{B}=T_{H}\delta S_{BH}+\left(\Omega_{h}-\Omega_{0}\right)\delta J_{B}+\Phi_{H}\delta Q_{B}\;, \end{array}$$

which is the first law of thermodynamics shown in Equation (7). Similar to Equation (34), this expression (in particular, the normalized energy) was obtained using the equations of motion without introducing any thermal information. Therefore, the charged particle absorption process satisfies the second law of thermodynamics and changes the black hole under the first law of thermodynamics.

Note that the laws of thermodynamics expressed by Equations (34) and (36) can also be considered in limits a→0 and Q→0. In vanishing limit *a*, the metric is equal to that of the Sen black hole, and $E_{0}$ = 0. This case represents the absorption of a charged particle by a charged black hole, and Equations (34) and (36) are still valid because this spacetime does not include coordinate rotation. Hence, the correction by $E_{0}$ is not needed. In addition, in vanishing limit *Q*, the black hole becomes a Kerr–(A)dS black hole, and Equations (34) and (36) are also valid. Finally, in vanishing limit Λ,Q, the metric reduces to the Kerr black hole. In this case, the laws of thermodynamics that we obtained also apply without the correction $E_{0}$. This is related to the third law of thermodynamics for the Kerr black hole case [86].

In addition, the location of the cosmological horizon infinitesimally changes by

(37) $$\begin{array}{c} \delta r_{c}=-\frac{\Delta_{Mc}P_{Lh}+\Delta_{Jc}}{\Delta_{Dc}}L-\frac{\Delta_{Mc}P_{Qh}+\Delta_{Qc}}{\Delta_{Dc}}e-\frac{\Delta_{Mc}P_{Rh}}{\Delta_{Dc}}\left|p^{r}\right|\;, \end{array}$$

where

(38) $$\begin{array}{c} \Delta_{Dc}=-\frac{2}{3}\left(3M+r_{c}\left(-3+a^{2}\Lambda +2r_{c}^{2}\Lambda \right)\right)\;,\;\Delta_{Qc}=2Q\Xi \;, \end{array}$$

$$\begin{array}{c} \Delta_{Jc}=-\frac{2a\Xi \left(-2Q^{2}\Lambda -3\Xi +r_{c}\Lambda \left(4M+r_{c}\Xi \right)\right)}{3M}\;, \end{array}$$

$$\begin{array}{c} \Delta_{Mc}=\frac{2\Xi \left(-3Mr_{c}\Xi +a^{2}(-2Q^{2}\Lambda -3\Xi +r_{c}\Lambda \left(4M+r_{c}\Xi \right)\right)}{3M}\;. \end{array}$$

Thus, the change of the black hole affects the cosmological horizon in a way that depends on the outer horizons, $r_{h}$.

## 4. Validity of Cosmic Censorship Conjecture in Extremal Black Hole

We will now investigate the validity of the cosmic censorship conjecture for an extremal KNdS black hole under charged particle absorption. An extremal black hole has maximum angular momentum and electric charge for a given mass. Thus, we can prove the validity of the cosmic censorship conjecture by showing that the overspinning or overcharging of the black hole is impossible. The change of the black hole is studied using the normalized energy in Equation (30), which is consistent with the laws of thermodynamics in Equations (34) and (36). The outer horizon of the extremal black hole is located at the minimum point $r_{e}=r_{h}$ of function $\Delta_{r}$. The extremal conditions for the initial black hole are:

(39) $$\begin{array}{cc} \Delta_{h} & =\Delta_{r}{|}_{r=r_{h}}=0\;,\;\Delta_{Dh}=0\;,\;\Delta_{DDh}=\frac{\partial^{2}\Delta_{h}}{\partial r_{h}^{2}}=2-\frac{2}{3}\left(6r_{h}^{2}+a^{2}\right)>0\;, \end{array}$$

where the second condition is equivalent to that of the extremal black hole with zero temperature, and the third condition indicates that the extremal point of $\Delta_{r}$ is a minimum. The aforementioned functions depend on parameters such as *M*, *a*, and *Q*. Thus, they will be changed when the black hole absorbs a charged particle. The change of function $\Delta_{r}$ depends on the variations of the parameters related to the energy and charges of the particle in Equation (27), and can be complicated. However, the validity of the cosmic censorship can be tested by simply observing the minimum value of the function $\Delta_{r}$ after the absorption, such that the test is straightforward. After particle absorption, the minimum value is shifted upward or downward. Hence, a negative minimum of the function implies the existence of the outer horizon of the black hole. Thus, the cosmic censorship conjecture is valid. However, a positive minimum would imply that there is no outer horizon, thus making the spacetime a naked singularity because there is no solution to $\Delta_{r}=0$ for an upward shift. After the absorption of the particle, the position of the minimum will be slightly shifted to $r_{h}+\delta r_{e}$, such that the extremal condition of the function $\Delta_{r}$ is changed after the particle absorption. At the displaced minimum position, $r_{h}+\delta r_{e}$,

(40) $$\begin{array}{c} \delta \Delta_{Dh}=\frac{\partial \Delta_{Dh}}{\partial M_{B}}\delta M_{B}+\frac{\partial \Delta_{Dh}}{\partial J_{B}}\delta J_{B}+\frac{\partial \Delta_{Dh}}{\partial Q_{B}}\delta Q_{B}+\frac{\partial \Delta_{Dh}}{\partial r_{h}}\delta r_{e}=0\;, \end{array}$$

where

(41) $$\begin{array}{c} \Delta_{DMh}=\frac{\partial \Delta_{Dh}}{\partial M_{B}}=\frac{2\Xi \left(-3M\Xi +2a^{2}\Lambda \left(2M+r_{h}+\Xi \right)\right)}{3M}\;, \end{array}$$

$$\begin{array}{c} \Delta_{DJh}=\frac{\partial \Delta_{Dh}}{\partial J_{B}}=\frac{4a\Lambda \Xi (2M+r_{h}\Xi )}{3M}\;,\;\Delta_{DQh}=\frac{\partial \Delta_{Dh}}{\partial Q_{B}}=0\;. \end{array}$$

Thus, by inserting Equation (30), we determine that the minimum is shifted from $r_{e}$ by

(42) $$\begin{array}{c} \delta r_{e}=-\frac{\Delta_{DMh}P_{Lh}+\Delta_{DJh}}{\Delta_{DDh}}L-\frac{\Delta_{DMh}P_{Qh}}{\Delta_{DDh}}e-\frac{\Delta_{DMh}P_{Rh}}{\Delta_{DDh}}\left|p^{r}\right|\;. \end{array}$$

The change in the position of the minimum depends on the momentum and the electric charge of the particle, such that the minimum may be shifted in the positive or negative direction of the radial coordinate by the charged particle. However, the value of function $\Delta_{r}$ at the shifted position of the minimum is also modified because of the particle charges. The change in the minimum value of function $\Delta_{r}$ is obtained from

(43) $$\begin{array}{cc} \delta \Delta_{h} & =\frac{\partial \Delta_{h}}{\partial M_{B}}\delta M_{B}+\frac{\partial \Delta_{h}}{\partial J_{B}}\delta J_{B}+\frac{\partial \Delta_{h}}{\partial Q_{B}}\delta Q_{B}+\frac{\partial \Delta_{h}}{\partial r_{h}}\delta r_{e}\;,\\ & =\Delta_{Mh}\delta M_{B}+\Delta_{Jh}\delta J_{B}+\Delta_{Qh}\delta Q_{B}+\Delta_{Dh}\delta r_{e}=0\;, \end{array}$$

where $\Delta_{Dh}=0$ from the initial condition in Equation (39). By using coefficients in Equations (30) and (32), we can then obtain the minimum value of function $\Delta_{r}$ at the shifted position:

(44) $$\begin{array}{c} \Delta_{r}\left(r_{e}+\delta r_{e}\right)=-\frac{2\rho_{e}^{2}\Xi }{r_{e}}\left|p^{r}\right|<0\;,\;\rho_{e}^{2}=\rho^{2}{|}_{r=r_{e}}\;, \end{array}$$

where only the coefficient of the radial momentum $|p^{r}|$ remains, and others become zero. This minimum value is negative and independent of the angular momentum and electric charge of the particle. Thus, it will always be negative after particle absorption. This also implies that there are two solutions around the minimum corresponding to the inner and outer horizons of the black hole. As a result, the extremal black hole turns nonextremal with two horizons (in particular, the outer horizon still exists after particle absorption, and hence the cosmic censorship conjecture is true). These results imply that the mass of the extremal black hole increases more than the angular momentum and electric charge under the absorption of a charged particle, as shown in Equation (44). Thus, the black hole cannot be overspun or overcharged beyond extremality, and the horizon still covers its curvature singularity. Therefore, the cosmic censorship conjecture is valid for the KNdS black hole under charged particle absorption.

In addition, note that the cosmic censorship conjecture is also true in vanishing limit *a* or *Q* because the laws of thermodynamics were found to be valid in these cases. The new minimum of $\Delta_{r}$ given by Equation (44) does not depend on the charge or angular momentum of the particle. Therefore, it becomes negative for any particle in a KNdS black hole. Note that our result also holds for arbitrary (positive, negative, or zero) values of the cosmological constant.

We can also investigate the change in the cosmological horizon for the extremal black hole by using a similar method. The location of the cosmological horizon does not correspond to an extremal point of $\Delta_{r}$. Therefore,

(45) $$\begin{array}{c} \Delta_{c}=\Delta_{r}{|}_{r=r_{c}}=0\;,\;\partial_{r}\Delta_{c}=\frac{\partial \Delta_{r}}{\partial r}{|}_{r=r_{c}}<0\;. \end{array}$$

After particle absorption, the change in the value of function $\Delta_{r}$ at $r_{c}$ is

(46) $$\begin{array}{c} \delta \Delta_{c}=\left(\Delta_{Jc}+P_{Lh}\Delta_{Mc}\right)L+\left(\Delta_{Qc}+P_{Qh}\Delta_{Mc}\right)e+P_{Rh}\Delta_{Mc}\left|p^{r}\right|\;, \end{array}$$

where $\delta \Delta_{c}$ still depends on the angular momentum and the electric charge of the particle. As a result, the change (i.e., $\delta \Delta_{c}$) can be positive or negative. This differs from the change of the minimum value in Equation (44). The singularity is located inside the horizon of the black hole. Hence, there is no reason for the cosmological horizon to move in a specific direction because there is no curvature singularity beyond the cosmological horizon. Therefore, the cosmic censorship conjecture plays an important role in the behavior of black hole horizons.

## 5. Summary

We studied herein the laws of thermodynamics and found that the cosmic censorship conjecture is valid in the extremal KNdS black hole under charged particle absorption. The extremal black hole has maximum spin and charge parameters for a given mass. Therefore, we investigated whether the extremal black hole can be overspun or overcharged by a charged particle entering the black hole. In this case, the conserved quantities of the black hole change as much as those of the particle. Thus, the relation between the conserved quantities of the particle determines the relation between the changes in the black hole charges. We focused on the laws of thermodynamics on the outer horizon to obtain the correct equations of motion. To satisfy the second law of thermodynamics, the energy of the particle was redefined to *E* by removing the dependency on the rotating directions by using reference energy $E_{0}$. The entropy of the black hole calculated from this redefined energy was then shown to monotonically increase. Based on the change in the entropy, we could rewrite the redefined energy of the particle to obtain the first law of thermodynamics on the outer horizon. We applied the redefined energy of the particle to the extremal black hole to test the cosmic censorship conjecture. The change of the black hole can be simply inferred from that of the minimum value in the function $\Delta_{r}$. The function always has a negative minimum under charged particle absorption. Thus, the extremal black hole turns nonextremal and still has an outer horizon, implying that the mass of the extremal black hole increases more than the angular momentum and the electric charge. Therefore, the cosmic censorship conjecture is valid for the extremal KNdS black hole. Note that our choice for reference energy $E_{0}$ is supported by the second law of thermodynamics as well as the agreement of the cosmic censorship conjecture. The reference energy $E_{0}$ = 0 for the asymptotically flat case. Hence, our modification for the laws of thermodynamics is consistent with the results of the Kerr–Newman black hole.

## Appendix A. Violation of the Cosmic Censorship Conjecture

If we use Equation (17) to test the cosmic censorship conjecture without $E_{0}$, we find that it violates not only the second law of thermodynamics, but also the cosmic censorship conjecture. Under the same initial conditions of Equation (39), the change in the minimum value of the function $\Delta_{r}$ is obtained as

(47) $$\begin{array}{cc} \delta \Delta_{h} & =\frac{\partial \Delta_{h}}{\partial M_{B}}\delta M_{B}+\frac{\partial \Delta_{h}}{\partial J_{B}}\delta J_{B}+\frac{\partial \Delta_{h}}{\partial Q_{B}}\delta Q_{B}+\frac{\partial \Delta_{h}}{\partial r_{h}}\delta r_{e}\;,\\ & =\Delta_{Mh}\delta M_{B}+\Delta_{Jh}\delta J_{B}+\Delta_{Qh}\delta Q_{B}+\Delta_{Dh}\delta r_{e}=0\;, \end{array}$$

where now we will insert Equation (19) instead of Equation (30) into to $\delta M_{B}$. Then, we find that

(A2) $$\begin{array}{c} \delta \Delta_{h}=\left(\Delta_{Jh}+\frac{a\Delta_{Mh}\Xi }{r_{h}^{2}+a^{2}}\right)L+\left(\Delta_{Qh}+\frac{Q\Delta_{Mh}r_{h}}{r_{h}^{2}+a^{2}}\right)e+\frac{\Delta_{Mh}\rho_{h}^{2}}{r_{h}^{2}+a^{2}}\left|p^{r}\right|\;, \end{array}$$

where the coefficients of $\delta J_{B}$ and $\delta Q_{B}$ cannot be reduced to zero. This is noticeably different from Equation (44). Using Equation (A2), even if we set the radial momentum $p^{r}$ to zero, the minimum value of the function $\Delta_{r}$ can become positive by a proper choice of *L* and *e*, so the horizon of the black hole can disappear by upon the particle absorption. Therefore, the cosmic censorship conjecture is violated.
